# Erectile Dysfunction Is Associated With Excessive Growth Hormone Levels in Male Patients With Acromegaly

**DOI:** 10.3389/fendo.2021.633904

**Published:** 2021-05-04

**Authors:** Zhengyuan Chen, Xiaoqing Shao, Min He, Ming Shen, Wei Gong, Meng Wang, Yichao Zhang, Wenjuan Liu, Zengyi Ma, Zhao Ye, Yongning Lu, Nianqin Yang, Shanwen Chen, Lydia Hu, Yiming Li, Yongfei Wang, Yao Zhao, Zhaoyun Zhang

**Affiliations:** ^1^ Department of Neurosurgery, Huashan Hospital, Fudan University, Shanghai, China; ^2^ Shanghai Clinical Medical Center of Neurosurgery, Shanghai, China; ^3^ Shanghai Key Laboratory of Brain Function and Regeneration, Huashan Hospital, Fudan University, Shanghai, China; ^4^ Department of Endocrinology and Metabolism, Huashan Hospital, Shanghai Medical College, Fudan University, Shanghai, China; ^5^ Reproductive Medicine Center, Zhongshan Hospital, Fudan University, Shanghai, China; ^6^ Department of Urology, Zhongshan Hospital, Fudan University, Shanghai, China; ^7^ Department of Urology, Huashan Hospital, Fudan University, Shanghai, China; ^8^ Department of Biochemistry and Cellular and Molecular Biology, College of Arts and Sciences, The University of Tennessee, Knoxville, Knoxville, TX, United States

**Keywords:** acromegaly, erectile dysfunction, NPTR assessment, GH suppression, sexual dysfunction

## Abstract

**Objective:**

To determine the risk factors for erectile dysfunction (ED) in male patients with acromegaly and to prospectively investigate the short-term changes of erectile function after surgery or medical treatment.

**Methods:**

Sixty-three male patients were subjected to nocturnal penile tumescence and rigidity (NPTR) test for the evaluation of erectile function. The measurement of serum nitric oxide (NO) was also performed. Twenty-seven patients were re-evaluated by NPTR after surgery or long-term somatostatin analogues (SSA) treatment.

**Results:**

Twenty-two patients (34.9%) had ED. Patients with ED showed higher random GH (17.89 [10.97-44.19] μg/L *vs* 11.63 [4.31-28.80] μg/L, *p* = 0.020) and GH nadir (GHn) (10.80 [6.69-38.30] μg/L *vs* 8.76 [3.62-18.19] μg/L, *p* = 0.044) during oral glucose tolerance test (OGTT). The NO levels of ED patients were lower than non-ED patients (9.15 [5.58-22.48] μmol/L *vs* 16.50 [12.33-31.78] μmol/L, *p* = 0.012). After treatment, patients who present improvement in erectile function showed lower post-GHn (0.07 [0.03-0.12] ng/ml *vs* 1.32 [0.09-3.60] ng/ml, *p* = 0.048) and post-IGF-1 index (1.03 ± 0.38 *vs* 1.66 ± 0.95, *p* = 0.049). The multivariate analysis indicated post-GHn was still associated with the improvement of erectile function after correction of other covariates (OR: 0.059, 95% CI: 0.003-1.043, *p* = 0.053).

**Conclusions:**

Excessive GH is related to ED in male patients with acromegaly. GH normalization after treatment is beneficial for short-term erectile function recovery.

## Introduction

Acromegaly is a rare, chronic disorder characterized by the hypersecretion of growth hormone (GH) and insulin-like growth factors (IGF-1), primarily caused by a GH-secreting pituitary adenoma (1). Acromegaly has considerable burdens in regards to complications and coexisting morbidities including diabetes, hypertension, sleep apnea and hyperlipemia (2). In terms of gonadal disturbance, previous studies have shown the high prevalence of erectile dysfunction (ED) in male patients with acromegaly, ranging from 42.1% to 62.7% (3, 4). ED can be broadly classified into organic (physical conditions), psychologic (life stresses) and relational (unsatisfied couple relationship) causes (5–8). However, to date, the causes for ED in patients with acromegaly remains controversial. GH excess per se, hypogonadism caused by tumor compression or other multiple factors have been speculated as potential causes (9–11).

Previously we, using international index of erectile function-5 (IIEF-5) questionnaire, reported that ED was highly prevalent among male patients with acromegaly in China and may be related to decreased nitric oxide (NO) induced by excessive growth hormone (GH) (4). However, there remains the question of whether the subjective questionnaire, reflecting perception of erectile function, is an adequate and appropriate reflection of physiological capacity and also it appears that subjective personal compliance might influent the efficacy of questionnaires especially in a relatively conservative country like China. Moreover, it was unclear whether or not ED would be improved after surgical tumor resection or medical treatment. Thus, we were inspired to apply a more accurate and objective instrument to quantitatively assess erectile function in male patients with acromegaly and to prospectively investigate the short-term changes of erectile function after surgery or medical treatment.

Men with normal erectile function experience 4–6 episodes of involuntary nocturnal erections, which last for 20–50 minutes during a 6–8 hour sleep cycle (12). Nocturnal penile tumescence and rigidity (NPTR) test is one of the most reliable tools to diagnose ED and also has been recommended as the gold standard for distinguishing between psychogenic and organic ED (13). However, NPTR has not yet been used to evaluate ED in male patients with acromegaly.

In this study we used Rigiscan, a NPTR assessment instrument, to evaluate ED in male patients with acromegaly and identify related risk factors. Furthermore, we prospectively investigated the short-term changes of erectile function after surgery or medical treatment.

## Subjects and Methods

### Subjects

From October 2016 to February 2020, 63 male patients with acromegaly were enrolled from a single hospital. The diagnosis of acromegaly was based on typical signs and symptoms of acromegaly, elevated insulin-like growth factors-1 (IGF-1) compared with the age-adjusted reference ranges, elevated GH levels not suppressible below 0.4 μg/L in response to 75 g oral glucose tolerance test (OGTT), and MRI evidence of pituitary adenoma. Data collection included demographics, life-style characteristics, and comorbidities. The physical examination of all patients was performed by experienced physicians. Patients were excluded if they had one of the following conditions: genital or spinal cord injuries, penile anomalies, prostate tumor, psychiatric disorders, and/or visual and hearing disturbances. Patients who had taken testosterone (or derivates) replacement therapy within 12 months were also excluded. This study was conducted in compliance with the ethical principles of the Declaration of Helsinki and approved by the ethical committee. Written informed consent was obtained from each subject prior to their participation. After baseline evaluation, 27 patients received either endoscopic transsphenoidal tumor resection or SSA treatment (long-acting octreotide, 30 mg per 4 weeks) and were arranged to Rigiscan re-evaluation.

### Biochemical Measurements

Random GH, GH nadir (GHn) after 75 g OGTT, and IGF-1 levels were measured. GH was measured by a 2-site chemiluminescent immunometric assay (AutoDELFIA^®^ hGH, PerkinElmer Life and Analytical Sciences, Wallac Oy, Finland). IGF-1 was measured using an Immulite 2000 solid-phase, enzyme-labeled chemiluminescent immunometric assay (Siemens Healthcare Diagnostic Products Limited, UK). The IGF-1 index was calculated as the ratio of the IGF-1 level/the upper limit of age-adjusted reference ranges (14). The criteria of endocrine remission was defined as the reduction of IGF-1 levels to age-adjusted normal ranges, random GH levels to<1.0 μg/L and GHn levels <0.4 μg/L after OGTT (15). Other adenohypophyseal hormones including thyroid-stimulating hormone (TSH), free thyroxine (FT4), morning free cortisol, follicle-stimulating hormone (FSH), luteinizing hormone (LH), total testosterone, and prolactin (PRL) were measured. The measurement of serum NO levels was performed to assess endothelium function using the Nitrate/Nitrite Colorimetric Assay kit (Cayman, Ann Arbor, MI).

### MRI Studies

Spin-echo sequence T1-weighted pituitary MRI examinations before and after intravenous gadolinium administration were performed for all patients using a 3.0-T whole body scanner (General Electric Medical Systems, Milwaukee, MI). Adenomas that measured less than 10mm in the largest dimension were classified as microadenomas, while larger tumors were considered as macroadenomas. The tumor volume was calculated according to the equation developed by Di Chiro and Nelson (Tumor volume = length * height * width * π/6) (16).

### Nocturnal Penile Tumescence and Rigidity (NPTR) Assessment

The NPTR assessment was evaluated by trained physicians using the Rigiscan Plus monitor (Timm Medical Technologies, Inc. Minneapolis, MN, USA; a division of Plethora Solutions Holdings, Ltd., UK) as previously described (12). Two string loops were placed at the tip and the base of penis, respectively, to record rigidity and tumescence data for two consecutive nights. The first night was considered as an adaptation night, while measurements from the second night were used for analysis. Patients with sleep duration less than 5 hours per night were excluded. Patients were recommended to avoid the intake of psychoactive substances such as alcohol, caffeine, sleeping pills or selective phosphodiesterase type 5 inhibitors (PDE5-Is), for 1 week prior to the assessment. All data was then analyzed with the Rigiscan Plus software version 4.0.

In this study, the primary outcome for erectile function evaluation was the duration of 60% or greater tip rigidity. According to the guideline, the presence of an erection with tip rigidity greater than 60%, lasting at least 10 minutes, would be defined as a normal erection. Otherwise, it would be defined as ED (13, 17). Rigiscan also recorded several other parameters including the total number of qualified erections (per night), duration of erection (min), average event rigidity (%), as well as tumescence (%) at the tip and base of penis, which have been considered as secondary outcomes for erectile function evaluation.

### Statistical Analysis

Continuous variables were expressed as the mean ± standard deviation (SD) or median with interquartile range (non-normally distributed data). The comparison between two continuous variables was tested either by the Student t test or the Wilcoxon test (data not normally distributed). Paired t-test was adopted to compare the valuables before and after the treatment. Categorical variables were presented as percentages and were analyzed either by the Pearson’s chi-square test or the Fisher’s exact test for comparison. The multivariable logistic regression model was constructed to identify potential independent risk factors. Correlation was tested either with the Pearson correlation coefficients or the Spearman Rank correlation coefficients. Statistical evaluations were performed using the SPSS 22.0 software. *P*<0.05 was considered statistically significant.

## Results

### Patients Characteristics

A total of 63 male patients with acromegaly were enrolled in this study. The detailed clinical characteristics of study population are displayed in **Table 1**. The mean age of 63 patients with acromegaly enrolled was 36.3 ± 8.6 years; mean duration of disease was 5.7 ± 4.5 years. 18 (26.9%), 4 (5.9%) and 12 (17.9%) patients had previous histories of surgery, radiotherapy or medical therapy, respectively.

**Table 1:** Clinical baseline characteristics of 63 male patients with acromegaly.

N = 63	
Age (years)	36.3 ± 8.6
BMI^a^ (kg/m^2^)	27.62 ± 3.79
Disease duration (years)	5.7 ± 4.5
Macroadenoma	32 (50.7%)
Tumor volume (cm^3^)	4.16 ± 0.97
Hypertension	15 (22.4%)
Diabetes mellitus	25 (37.3%)
OSAHS^b^	6 (9.5%)
Coronary artery disease	4 (5.9%)
Smoking	13 (19.4%)
Antihypertensive medication	12 (17.9%)
Previous surgery	18 (26.9%)
Previous pituitary radiotherapy	4 (5.9%)
Previous therapy with SSA^c^	12 (17.9%)
Random GH^d^ (μg/L)	13.70 [5.67-32.50]
GHn (μg/L)	10.17 [4.38-24.92]
IGF-1^e^ index	2.60 ± 0.81
Total testosterone (nmol/L)	6.94 ± 4.39
FSH^f^ (IU/L)	6.58 ± 4.82
LH^g^ (IU/L)	4.14 ± 2.66
PRL^h^ (ng/mL)	16.37 [8.02-34.78]
Free cortisone (μg/dl)	10.22 ± 4.63
TSH^i^ (mIU/L)	1.01 [0.71-1.87]
FT_4_^j^ (pmol/L)	15.52 ± 3.18

BMI^a^, body mass index; OSAHS^b^, obstructive sleep apnea-hypopnea syndrome; SSA^c^, somatostatin analogues; GH^d^, growth hormone; IGF-1^e^, insulin like growth factor-1; FSH^f^, follicle-stimulating hormone; LH^g^, luteinizing hormone; PRL^h^, prolactin; TSH^i^, thyroid-stimulating hormone; FT_4_^j^, free thyroxine.

### Baseline NPTR Assessment and NO Measurement

Based on the diagnosis criteria, 22 (34.9%) patients were diagnosed with ED. The prevalence of ED in different age groups was 29.4% (20-29 years), 47.8% (30-39 years), and 26.1% (>40 years). Between patients with and without ED, there were no differences regarding previously reported ED risk factors including total testosterone levels (6.09 ± 4.18 *vs* 7.41 ± 4.55 nmol/L, *p* = 0.377), prolactin levels (20.44 [13.72-40.10] ng/ml *vs* 13.78 [7.29-50.9] ng/ml, *p* = 0.466), age (35.8 ± 7.6 *vs* 37.0 ± 9.1 years, *p* = 0.624), BMI (27.85 ± 4.40 *vs* 27.64 ± 3.50 kg/m^2^, *p* = 0.845), percentage of hypertension (22.7% *vs* 24.4%, *p* = 0.883), percentage of diabetes mellitus (27.3% *vs* 41.0%, *p* = 0.265), percentage of coronary artery disease (4.5% *vs* 4.9%, *p* = 0.953), and smoking (22.7% *vs* 17.1%, *p* = 0.586). In addition, no differences were observed between 2 groups in terms of previous treatment history, disease duration, tumor volume, free cortisone, TSH, FT_4_, and IGF-1 index, which were demonstrated in **Table 2**.

**Table 2:** Clinical characteristics of ED^a^ and non-ED patients.

	Non- ED (n = 41)	ED (n = 22)	*P* value
Age (years)	37.0 ± 9.1	35.8 ± 7.6	0.624
Disease duration (years)	5.3 ± 3.8	6.9 ± 5.5	0.193
BMI^b^ (kg/m^2^)	27.64 ± 3.50	27.85 ± 4.40	0.845
Macroadenoma	20 (48.8%, 20/41)	12 (54.5%, 12/22)	0.663
Tumor volume (cm^3^)	3.73 ± 1.09	5.21 ± 2.03	0.492
Hypertension	10 (24.4%, 10/41)	5 (22.7%, 5/22)	0.883
Diabetes mellitus	17 (41.0%, 17/41)	6 (27.3%, 6/22)	0.265
OSAHS^c^	4 (9.8%, 4/41)	2 (9.1%, 2/22)	0.932
Coronary artery disease	2 (4.9%, 2/41)	1 (4.5%, 1/22)	0.953
Smoking	7 (17.1%, 7/41)	5 (22.7%, 5/22)	0.586
Previous surgery	8 (19.5%, 8/41)	8 (36.4%, 8/22)	0.143
Previous pituitary radiotherapy	2 (4.9%, 2/41)	2 (9.0%, 2/22)	0.513
On therapy with SSA^d^	6 (14.6%, 6/41)	4 (18.2%, 4/22)	0.713
Random GH^e^ (μg/L)	11.63 [4.31-28.80]	17.89 [10.97-44.19]	0.020*
GHn (μg/L)	8.76 [3.62-18.19]	10.80 [6.69-38.30]	0.044*
IGF-1^f^ index	2.70 ± 0.74	2.63 ± 0.81	0.679
Total testosterone (nmol/L)	7.41 ± 4.55	6.09 ± 4.18	0.377
FSH^g^ (IU/L)	6.41 ± 3.15	4.58 ± 2.73	0.033*
LH^h^ (IU/L)	4.12 ± 2.13	3.46 ± 2.70	0.063
PRL^i^ (ng/mL)	13.78 [7.29-50.9]	20.44 [13.72-40.10]	0.466
Free cortisone (μg/dl)	9.32 ± 4.39	9.70 ± 4.60	0.772
TSH^j^ (mIU/L)	0.91 [0.56-1.05]	1.46 [0.93-2.05]	0.201
FT_4_^k^ (pmol/L)	16.02 ± 3.35	14.34 ± 3.68	0.199

ED^a^, erectile dysfunction; BMI^b^, body mass index; OSAHS^c^, obstructive sleep apnea-hypopnea syndrome; SSA^d^, somatostatin analogues; GH^e^, growth hormone; IGF-1^f^, insulin like growth factor-1; FSH^g^, follicle-stimulating hormone; LH^h^, luteinizing hormone; PRL^i^, prolactin; TSH^j^, thyroid-stimulating hormone; FT_4_^k^, free thyroxine.

P* < 0.05.

**Figure 1:**
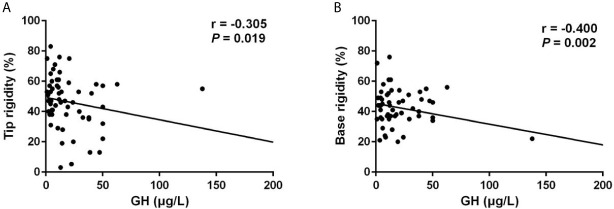
Random GH levels were negatively correlated with the Rigiscan parameters. **(A)** Random GH levels were negatively correlated with average tip rigidity (*r* = -0.305, *p* = 0.019). **(B)** Random GH levels were negatively correlated with the average base rigidity (*r* = -0.400, *p* = 0.002).

**Figure 2:**
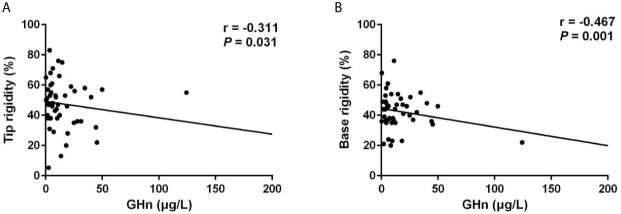
GHn levels were negatively correlated with the Rigiscan parameters. **(A)** GHn levels were negatively correlated with the average tip rigidity (*r* = -0.311, *p* = 0.031). **(B)** GHn were negatively correlated with the average base rigidity (*r* = -0.467, *p* = 0.001).

Notably, patients with ED presented significantly higher random GH levels (17.89 [10.97-44.19] μg/L *vs* 11.63 [4.31-28.80] μg/L, *p* = 0.020), and GHn levels (10.80 [6.69-38.30] μg/L *vs* 8.76 [3.62-18.19] μg/L, *p* = 0.044). Multivariable logistic regression analysis demonstrated that the independent risk factor for ED was GHn (OR: 4.459, 95% CI: 1.008-19.733, *p* = 0.049) after correction of covariates including IGF-1 index and total testosterone (**Table 3**). The correlation analysis showed that random GH was negatively associated with the number of erection events (r = -0.259, *p* = 0.046), average tip rigidity (r = -0.343, *p* = 0.007), increasement of tip circumference (r = -0.259, *p* = 0.045), and average base rigidity (r = -0.435, *p* = 0.001). GHn was negatively correlated with average tip rigidity (r = -0.324, *p* = 0.023), as well as average base rigidity (r = -0.462, *p* = 0.001). To verify the reliability of the correlation analysis, we excluded the outlier in the following analysis. It showed that random GH is still negatively correlated with average tip rigidity (r = -0.305, *p* = 0.019, **Figure 1A**) and base rigidity (r = -0.400, *p* = 0.002, **Figure 1B**) after excluding the outlier. Also, there is still a negative association between GHn and average tip rigidity (r = -0.311, *p* = 0.031, **Figure 2A**) and base rigidity (r = -0.467, *p* = 0.001, **Figure 2B**). We observed no significant correlation between total testosterone levels and any Rigiscan parameter (**Supplementary Table 1**). In addition, there was a positive correlation between FT_4_ levels (r = 0.338, *p* = 0.007) and the number of erections. Free cortisone levels were negatively correlated with the number of erections (r = -0.295, *p* = 0.021) and erection duration (r = -0.351, *p* = 0.006). The full correlation analysis between pituitary hormones and Rigiscan parameters was showed in **Supplementary Table 1**.

**Table 3:** Multivariate analysis between ED^a^ and non-ED patients in the baseline.

	B	S.E.	Wald	Sig.	Exp(B)	95% C.I.for EXP(B)	
						Lower	Upper
Log pre-GHn^b^	1.495	0.759	3.881	0.049*	4.459	1.008	19.733
Log pre-IGF-1^c^ index	-1.745	2.660	0.430	0.512	0.175	0.001	32.098
Log pre-Testosterone	0.175	0.864	0.041	0.839	1.191	0.219	6.474
Constant	-1.567	1.338	1.372	0.241	0.209		

ED^a^, erectile dysfunction; GHn^b^, nadir growth hormone after oral glucose tolerance test; IGF-1^c^, insulin like growth factor-1.

P* < 0.05.

**Figure 3:**
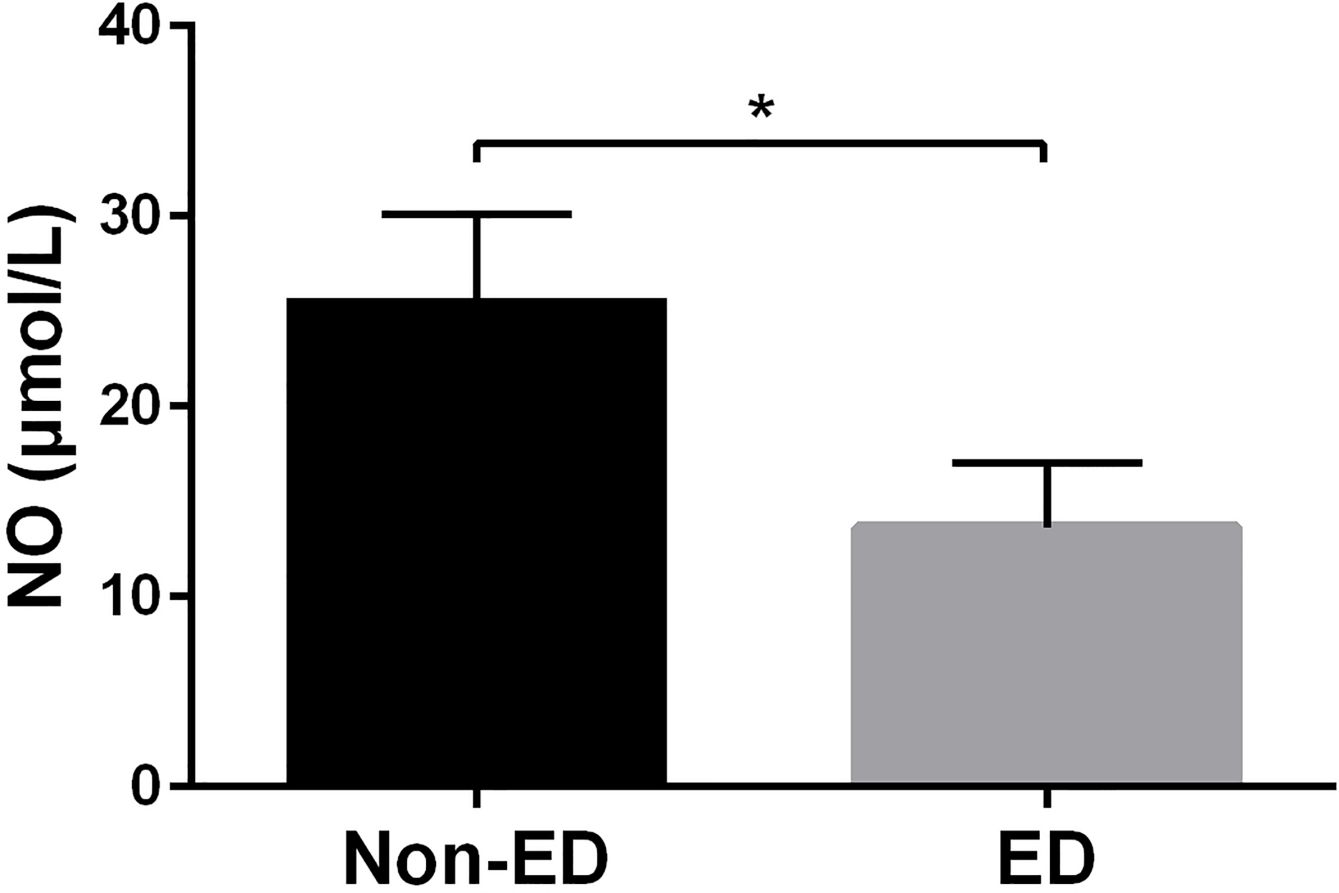
NO levels were significantly decreased in patients with ED. Patients with ED had significantly lower NO levels than non-ED patients (9.15 [5.58-22.48] μmol/L *vs* 16.50 [12.33-31.78] μmol/L, *p* = 0.012). * means p-value < 0.05.

NO, which is released from the penile endothelial cells, is the primary neurotransmitter associated with normal erection. NO reduction play significant roles in the pathophysiology of ED (18). As shown in **Figure 3**, we found that NO levels of ED patients were significantly lower compared to non-ED patients (9.15 [5.58-22.48] μmol/L *vs* 16.50 [12.33-31.78] μmol/L, *p* = 0.012).

### NPTR Revaluation After Treatment

Of the 63 patients, 27 patients received re-evaluation of NPTR after endoscopic endonasal tumor resection (n = 23, 85.2%) or SSA treatment (n = 4, 14.8%). The mean follow-up period was 4.1 months. Among the 27 patients who receive Rigiscan re-examination, 8 patients have ED and the other 19 patients showed normal erectile function at baseline. Overall, there was modest improvement in duration of tip rigidity>60% (13.50 [5.25-19.25] min *vs* 16.50 [3.75-22.5] min, *p* = 0.283) and secondary outcomes including number of erections (6.16 ± 3.65 *vs* 6.16 ± 3.21, *p* = 1.000), erection duration (73.50 [48.75-113.50] min *vs* 77.50 [48.38-93.25] min, *p* = 0.957), average rigidity (Tip: 41.76 ± 18.59% *vs* 48.48 ± 16.01%, *p* = 0.194; Base: 41.12 ± 12.29% *vs* 41.48 ± 9.57%, *p* = 0.893) and change of tumescence (Tip: 28.40 ± 13.28% *vs* 34.76 ± 13.35%, *p* = 0.101; Base: 40.80 ± 11.35% *vs* 44.56 ± 7.21%, *p* = 0.119) compared to baseline (**Table 4**). Furtherly, we subdivided 27 patients into 2 groups according to whether an improved duration of tip rigidity>60% was presented after treatment. The univariate analysis showed patients of improvement group presented significant higher percentage of GHn normalization (88.9% *vs* 46.7%, *p* = 0.039), IGF-1 normalization (72.7% *vs* 25.0%, *p* = 0.040) other than testosterone normalization (63.6% *vs* 43.8%, *p* = 0.440) after treatment (**Table 5**). Also, patients of improvement group showed significantly lower post-GHn (0.07 [0.03-0.12] ng/ml *vs* 1.32 [0.09-3.60] ng/ml, *p* = 0.048) and post-IGF-1 index (1.03 ± 0.38 *vs* 1.66 ± 0.95, *p* = 0.049) compared to non-improvement group (**Table 5**). The multivariate analysis showed that post-GHn is weakly associated with the improvement of erectile function after correction of covariates including post-IGF1 index and post-testosterone (OR: 0.102, 95% CI: 0.008-1.312, *p* = 0.080, **Table 6**). Finally, we performed correlation analysis in order to determine the association of erectile function recovery with hormone changes. The correlation analysis showed that the improvement of duration of tip rigidity>60% was correlated with GHn reduction% (*r* = 0.397, *p* = 0.075), while it did not attain to statistical significance. There were no correlations between random GH reduction %, IGF-1 index reduction %, testosterone elevation % and change of Rigiscan parameters (**Table 7**).

**Table 4:** Pituitary hormones and Rigiscan measurements before and after treatment.

	Baseline	After treatment	*P* value
Random GH^a^ (μg/L)	12.63 [3.64-35.45]	0.44 [0.20-4.00]	0.001**
GHn(μg/L)	12.26 [3.14-27.05]	0.09 [0.04-2.37]	0.001**
IGF-1^b^ index	2.63 ± 0.92	1.42 ± 0.88	0.000**
Total testosterone(nmol/L)	6.34 ± 4.26	9.71 ± 4.62	0.000**
FSH^c^(IU/L)	7.55 ± 6.54	7.58 ± 4.84	0.954
LH^d^(IU/L)	4.51 ± 3.45	5.32 ± 3.25	0.081
PRL^e^(ng/mL)	17.60 [8.57-83.71]	6.88 [5.02-10.53]	0.028*
Free cortisone (μg/dl)	10.59 ± 5.26	9.46 ± 3.80	0.418
TSH^f^ (mIU/L)	0.955 [0.550-2.065]	1.350 [0.838-1.558]	0.898
FT_4_^g^ (pmol/L)	16.32 ± 3.51	17.87 ± 3.09	0.020*
**Rigiscan measurements**			
Duration of tip rigidity>60% (min)	13.50 [5.25-19.25]	16.50 [3.75-22.5]	0.283
Number of erections (per night)	6.16 ± 3.65	6.16 ± 3.21	1.000
Erection duration (min)	73.50 [48.75-113.5]	77.50 [48.38-93.25]	0.957
Average tip rigidity (%)	41.76 ± 18.59	48.48 ± 16.01	0.194
Change of tip tumescence (%)	28.40 ± 13.28	34.76 ± 13.35	0.101
Average base rigidity (%)	41.12 ± 12.29	41.48 ± 9.57	0.893
Change of base tumescence (%)	40.80 ± 11.35	44.56 ± 7.21	0.119

GH^a^, growth hormone; IGF-1^b^, insulin like growth factor-1; FSH^c^, follicle-stimulating hormone; LH^d^, luteinizing hormone; PRL^e^, prolactin; TSH^f^, thyroid-stimulating hormone; FT_4_^g^, free thyroxine; P* < 0.05, P** < 0.01.

**Table 5:** Univariate analysis between patients with and without improvement in erectile function after treatment.

	Improvement group	Non-improvement group	*P* value
GH normalization after treatment (%)	72.7%	43.8%	0.239
Post-GH (ng/ml)	0.44 [0.15-1.03]	2.02 [0.21-5.27]	0.236
GHn normalization after treatment (%)	88.9%	46.7%	0.039*
Post-GHn (ng/ml)	0.07 [0.03-0.12]	1.32 [0.09-3.60]	0.048*
IGF-1 normalization after treatment (%)	72.7%	25.0%	0.040*
Post-IGF index	1.03 ± 0.38	1.66 ± 0.95	0.049*
Testosterone normalization after treatment (%)	63.6%	43.8%	0.440
Post-Testosterone (nmol/L)	10.56 ± 5.36	9.07 ± 4.43	0.437

**Table 6:** Multivariate analysis between patients with and without improvement in erectile function after treatment.

	B	S.E.	Wald	Sig.	Exp(B)	95% C.I.for EXP(B)	
						Lower	Upper
Log post-GHn^a^	-2.278	1.301	3.067	0.080	0.102	0.008	1.312
Log post-IGF1^b^ index	4.571	4.651	0.966	0.326	96.687	0.011	879961.913
Log post-Testosterone	-1.396	3.614	0.149	0.699	0.247	0.000	295.130
Constant	5.446	7.976	0.466	0.495	231.934		

GHn^a^, nadir growth hormone after oral glucose tolerance test; IGF-1^b^, insulin like growth factor-1.

**Table 7:** Correlation analysis associated with the changes of Rigiscan parameters.

		△GH%	△GHn%	△IGF-1 index%	△Testosterone%
**△ **Duration of tip rigidity>60% (min)	*r*	0.103	0.397	0.231	0.011
	*P*	0.608	0.075	0.256	0.957
**△ **Number of erections (per night)	*r*	-0.189	-0.248	-0.128	-0.016
	*P*	0.346	0.279	0.553	0.565
**△ **Erection duration (min)	*r*	-0.207	-0.011	-0.135	-0.035
	*P*	0.301	0.962	0.512	0.861
**△ **Average tip rigidity (%)	*r*	0.120	0.059	0.090	-0.066
	*P*	0.552	0.800	0.063	0.744
**△ **Change of tip tumescence (%)	*r*	0.067	0.022	0.221	-0.072
	*P*	0.741	0.925	0.277	0.723
**△ **Average base rigidity (%)	*r*	-0.012	0.083	0.038	-0.166
	*P*	0.951	0.720	0.853	0.409
**△ **Change of base tumescence (%)	*r*	0.203	0.223	0.131	0.081
	*P*	0.309	0.331	0.523	0.687

## Discussion

ED is a disorder which causes substantially negative effects not only on the individualistic level, but also on the socialistic level (18, 19). It is estimated that the prevalence of ED ranges from 6%-64%, with an average prevalence of 30% (20). However, ED has rarely been thoroughly studied in patients with acromegaly (3, 4).

Our previous work using IIEF-5 questionnaire has demonstrated that ED is rather prevalent (62.7%) among male patients with acromegaly in China, even in patients younger than 40 years of age (4). However, there remains the question of whether the subjective questionnaire, reflecting perception of erectile function, is an adequate and appropriate reflection of physiological capacity and also it appears that subjective personal compliance might influent the efficacy of questionnaires especially in a relatively conservative country like China. Thus, we were inspired to apply a more accurate and objective instrument to quantitatively assess erectile function in male patients with acromegaly. In clinical practice, penile color doppler ultrasound (PCDU) is considered as the most appropriate way for differentiating vascular and nonvascular ED. However, PCDU is less reliable in detecting other type of ED like neurogenic ED or endocrinological ED, which is the main type of ED in this study (13). Moreover, incomplete smooth muscle relaxation due to high anxiety or sympathetic overtone might lead to false-positive results (21). In comparison, NPTR is relatively old and not completely standardized. However, NPTR is non-invasive, painless and rarely disturbed by patient’s emotional state (18). Thus, we adopted Rigiscan, a NPTR instrument which is currently recommended by Canadian Urological Association (CUA) and European Urological Association (EUA) for ED diagnosis, to evaluate erectile function in this study (13, 17).

As far as we know, this is the first study to investigate the impact of and extent of acromegaly on erectile function using NPTR assessment. In this cohort, we found that the prevalence of ED was 34.9%, which is notably higher than the prevalence of hypertension (22.4%), and comparable to the prevalence of diabetes mellitus (37.3%). Surprisingly, we observed that even among young patients (aged 20-29 years), 29.4% had ED, which contradicts the established impression that ED mainly affected men older than 40 years of age. Furthermore, as far as we are concerned, ED is no longer perceived as just sexual issues, but it is also defined as an early predictor of major cardiovascular diseases (22, 23). Therefore, in view of its clinical significance, ED screening should be emphasized as an indispensable part of the evaluation in patients with acromegaly.

Despite the development of ED has been linked to several risk factors by previous studies including aging, hypertension, diabetes mellitus, testosterone deficiency, and/or lifestyle factors such as smoking and obesity (24, 25). It is still controversial about the causes for ED in male patients with acromegaly. GH excess per se, hypogonadism caused by tumor compression or other risk factors have been speculated (9–11). In this study, no significant association was found between ED and smoking, obesity, age, hypertension, or diabetes mellitus. Regarding to testosterone level, previous studies reported that testosterone was important in enhancing sexual desire and maintaining adequate nocturnal erections (19, 26). Hirshkowitz et al. reported that nocturnal erections are of lower frequency, rigidity, duration in men with testosterone deficiency, but could not be improved by testosterone replacement (27). In this study, we also noticed that patients with ED demonstrated slightly lower testosterone levels compared to patients without ED. However, the difference does not attain to statistical significance, suggesting that testosterone may not be the primary risk factor for the development of ED in patients with acromegaly. What we found may not be accidental. ED is the most common complaint in male patients with prolactinoma. It is generally believed that hypogonadism induced by PRL excess is the primary cause of ED in men with prolactinoma (28). However, most but not all male patients have ED secondary to low testosterone levels (26). A prospective study conducted by Colao et al. demonstrated that ED still persisted in 43% of the patients despite the normalization of testosterone (29). Few studies have discussed the roles of thyroid hormone and cortisone in ED. Hypoactive sexual desire and ED were documented by Carani et al. in hypothyroidism (30). Krassas et al. also reported that ED can be reversed after thyroxine replacement in patients with hypothyroidism (31). Data regarding erectile function in patients with hypercortisolism is still not available.

NO reduction play significant roles in the pathophysiology of ED (18). In this study, patients with ED showed significantly higher GH levels (other than IGF-1) and lower NO production compared to patients with normal erectile function. Thus, we assume that the high prevalence of ED in acromegaly might be associated with NO reduction induced by GH excess. Previous researches have already reported that excessive GH might induce endothelial dysfunction and subsequent NO reduction. Anagnostis and Giacchetti et al. reported that patients with acromegaly had significantly lower levels of NO (32, 33). Notably, Becker et al. showed that GH can elicit dose-dependent relaxation of human corpus cavernosum strips *in vitro* through cyclic guanosine monophosphate (cGMP) stimulating activity and that serum GH concentration of intracavernous regions vary during the different stages of penile erection, suggesting that GH may directly influent penile erection (34). All of these evidences indicated that ED in patients with acromegaly may be associated with excessive GH per se.

Whether or not ED in acromegaly might be reversible after surgery or SSA treatment is still unclear. In this study, all of the Rigiscan parameters only showed modest improvement after treatment indicating the improvement of erectile function is relatively slow and might not ameliorate in short-term. However, we found that patients who presented improvement in erectile function showed significant higher percentage of GHn normalization after treatment and lower post-GHn levels. There was no significant difference in testosterone level between improvement group and non-improvement group. Moreover, the improvement of erectile function was weakly correlated with GHn reduction% suggesting that GH reduction, other than testosterone elevation, might exert the beneficial effect on the improvement of erectile function. What we found may not be occasional. The same situation has been noticed in patients with prolactinoma. From an open longitudinal study conducted by Rosa et.al, cabergoline treatment for prolactinoma resulted in significant improvement of Rigiscan parameters in patients who had prolactin normalization, even when testosterone levels were still low. Conversely, for patients who did not achieve prolactin normalization, there was only modest improvement in Rigiscan parameters compared to baseline (35).

Limitations of the study arise from the small sample size and the short follow-up, which might decrease the power and accuracy of statistical analysis. Colao et al. reported significantly improved sperm number and motility in men with acromegaly after surgery or Lanreotide treatment with a follow-up of 6 months, indicating the possibility that the effects of GH suppression on erectile function may require longer period to be evident (36).

## Conclusion

Our study showed that ED in male patients with acromegaly might be associated with excessive GH and that GH normalization is beneficial for short-term erectile function recovery. Further studies compromising larger sample size and longer follow-up are required to further investigate the effect of GH suppression on erectile function recovery.

## Data Availability Statement

The original contributions presented in the study are included in the article/**Supplementary Material**. Further inquiries can be directed to the corresponding author.

## Ethics Statement

The studies involving human participants were reviewed and approved by ethics committee, huashan hospital, Fudan university. The patients/participants provided their written informed consent to participate in this study.

## Author Contributions

ZC: Conceptualization, Methodology, Data curation, Writing-original draft. XS: Conceptualization, Methodology, Data curation, Writing-original draft. MH: Writing-review and editing. MS and WG: Data curation. MW: Methodology. YCZ: Software and statistics. ZM and ZY: Conceptualization. YNL and NY: Methodology. LH and YML: Ethics supervision and editing the original draft. YW: Writing-review and editing. YZ and ZZ: Conceptualization, Methodology, Data curation, Writing-original draft. All authors contributed to the article and approved the submitted version.

## Funding

National Natural Science Foundation of China (No. 81970716) National Natural Science Foundation of China (No. 81770840) National Science Fund for Distinguished Young Scholars (No. 81725011) Shanghai Hospital Development Center (No.SHDC12018X04) National High Technology Research and Development Program of China (No.2014AA020611) Natural Science Foundation and Major Basic Research Program of Shanghai (No.16JC1420100) Shanghai Municipal Science and Technology Major Project (No.2018SHZDZX01) National Project in Promoting the Diagnosis and Treatment of Major Diseases by MDT.

## Conflict of Interest

The authors declare that the research was conducted in the absence of any commercial or financial relationships that could be construed as a potential conflict of interest.
