# Supplementary material for: Erectile Dysfunction Is Associated With Excessive Growth Hormone Levels in Male Patients With Acromegaly
Source: Front Endocrinol (Lausanne). 2021 May 4;12:633904. doi: 10.3389/fendo.2021.633904 (PMC8129559; doi:10.3389/fendo.2021.633904)
Supplement: Supplementary file 1 [file Table_1.docx]

**Supplementary Table 1. Full correlation analysis between pituitary hormones and Rigiscan parameters.**

|  | | Duration of tip rigidity＞60% (min) | Number of erections | Erection duration (min) | Average tip rigidity (%) | Change of tip tumescence (%) | Average base rigidity (%) | Change of base tumescence (%) |
| --- | --- | --- | --- | --- | --- | --- | --- | --- |
| Random GH^a^ (μg/L) | r | -.182 | -.225 | -.132 | -.305^*^ | -.219 | -.400^**^ | -.155 |
|  | *P* | .167 | .086 | .318 | .019 | .095 | .002 | .240 |
| GHn (μg/L) | r | -.197 | -.212 | -.143 | -.311^*^ | -.262 | -.467^**^ | -.185 |
|  | *P* | .179 | .147 | .332 | .031 | .071 | .001 | .209 |
| IGF-1^b^ Index | r | .174 | -.018 | .089 | .119 | .050 | .037 | .097 |
|  | *P* | .184 | .890 | .501 | .366 | .702 | .776 | .463 |
| Testosterone (nmol/L) | r | -.008 | .067 | .107 | .149 | .149 | -.080 | .008 |
|  | *P* | .952 | .610 | .410 | .253 | .253 | .539 | .953 |
| FSH^c^（IU/L） | r | 0.172 | 0.158 | 0.122 | 0.206 | 0.181 | 0.211 | 0.059 |
|  | *P* | 0.186 | 0.224 | 0.350 | 0.111 | 0.162 | 0.102 | 0.651 |
| LH^d^（IU/L） | r | 0.239 | 0.119 | 0.236 | 0.145 | 0.369** | 0.061 | 0.117 |
|  | *P* | 0.064 | 0.360 | 0.067 | 0.263 | 0.003 | 0.638 | 0.369 |
| PRL^e^（ng/mL） | r | -0.009 | -0.095 | -0.095 | 0.159 | 0.031 | 0.107 | 0.138 |
|  | *P* | 0.944 | 0.471 | 0.472 | 0.226 | 0.815 | 0.414 | 0.294 |
| Free cortisone (μg/dl) | r | -0.006 | -0.291* | -0.348** | 0.223 | -0.044 | 0.211 | 0.122 |
|  | *P* | 0.964 | 0.024 | 0.007 | 0.087 | 0.738 | 0.105 | 0.352 |
| FT4^f^ (pmol/L) | r | 0.015 | 0.306* | 0.140 | 0.008 | -0.014 | 0.015 | -0.203 |
|  | *P* | 0.910 | 0.016 | 0.282 | 0.953 | 0.916 | 0.906 | 0.116 |

GH^a^ (growth hormone), IGF-1^b^ (insulin like growth factor-1), FSH^c^ (follicle-stimulating hormone), LH^d^ (luteinizing hormone), PRL^e^(prolactin), FT4 ^f^ (free thyroxine)

P*＜0.05, P**＜0.01
